# Fostering the Aesthetic Pleasure: The Effect of Verbal Description on Aesthetic Appreciation of Ambiguous and Unambiguous Artworks

**DOI:** 10.3390/bs11110144

**Published:** 2021-10-23

**Authors:** Emanuela Mari, Alessandro Quaglieri, Giulia Lausi, Maddalena Boccia, Alessandra Pizzo, Michela Baldi, Benedetta Barchielli, Jessica Burrai, Laura Piccardi, Anna Maria Giannini

**Affiliations:** 1 Department of Psychology, “Sapienza” University of Rome, 00185 Rome, Italy; alessandro.quaglieri@uniroma1.it (A.Q.); giulia.lausi@uniroma1.it (G.L.); maddalena.boccia@uniroma1.it (M.B.); alessandra.pizzo@uniroma1.it (A.P.); michela.baldi@uniroma1.it (M.B.); benedetta.barchielli@uniroma1.it (B.B.); jessica.burrai@uniroma1.it (J.B.); laura.piccardi@uniroma1.it (L.P.); annamaria.giannini@uniroma1.it (A.M.G.); 2 Cognitive and Motor Rehabilitation and Neuroimaging Unit, IRCCS Fondazione Santa Lucia, 00179 Rome, Italy; 3 Department of Clinical and Dynamic Psychology, “Sapienza” University of Rome, 00185 Rome, Italy

**Keywords:** perceptual ambiguity, global–local perception, visual aesthetic, portraits, aesthetic experience

## Abstract

Background: Aesthetic experience begins through an intentional shift from automatic visual perceptual processing to an aesthetic state of mind that is evidently directed towards sensory experience. In the present study, we investigated whether portrait descriptions affect the aesthetic pleasure of both ambiguous (i.e., Arcimboldo’s portraits) and unambiguous portraits (i.e., Renaissance portraits). Method: A total sample of 86 participants were recruited and completed both a baseline and a retest session. In the retest session, we implemented a sample audio description for each portrait. The portraits were described by three types of treatment, namely global, local, and historical descriptions. Results: During the retest session, aesthetic pleasure was higher than the baseline. Both the local and the historical treatments improved the aesthetic appreciation of ambiguous portraits; instead, the global and the historical treatment improved aesthetic appreciation of Renaissance portraits during the retest session. Additionally, we found that the response times were slower in the retest session. Conclusion: taken together, these findings suggest that aesthetic preference was affected by the description of an artwork, likely due to a better knowledge of the painting, which prompts a more accurate (and slower) reading of the artwork.

## 1. Introduction

The definition of aesthetic experience is still a highly controversial issue. Several authors describe aesthetic experience as intentional and non-spontaneous [1]; others discuss the interplay between cultural and biological evolution in the formation of aesthetic preference [2] and the modulation of the aesthetic value of an object by the context [3,4], so that the same subject defined as art/non-art or placed in a gallery/computer-processed results in a different aesthetic experience. Several studies [5,6] introduce the idea of an aesthetic mindset (i.e., a top-down orientation during the aesthetic evaluation of an object), while others state that aesthetic experience is a combination of both top-down and bottom-up processes, as per information processing [7,8,9].

In this regard, aesthetic experience can be described as a unique, affectively colored, self-transcending subject–object relation in which cognitive processing is felt to flow differently than during everyday experiences [10,11]. It is the result of a coordinated series of different cognitive processes that, in turn, activate different areas of the brain [12,13].

Chatterjee [14], inspired by visual neuroscience studies, developed a theoretical model of the cognitive and the affective processes implicated in visual aesthetic preference. More specifically, he proposed that a series of information processing steps follow one another during visual aesthetic experience: first, all the elementary visual characteristics of the artworks are processed in the same way as other visual objects; second, the attentional processes redirect the elaboration towards the salient visual properties, such as composition, color, and shape; third, the attentional network modulates the processing by allowing the content of the artwork (e.g., landscapes, portraits); fourth, some feedback and feed-forward processes, which link the attentional and attributional circuits, further improve the experience of visual stimuli; finally, emotional systems are also involved. Cela-Conde and colleagues [15] suggested that two distinct cognitive events occur during the aesthetic experience, which take place at different time span: first, there is a general evaluation of the aesthetic qualities (i.e., the perception of a visual stimulus as beautiful or not), defined as “aesthetic appreciation sensu stricto”; and then there is a subsequent assessment of details of the aesthetic experience (i.e., whether it is interesting or original), called as “aesthetic appreciation sensu lato”. Aesthetic appreciation sensu stricto occurs between 250 ms and 750 ms, whereas aesthetic appreciation sensu lato occurs between 1000 ms and 1500 ms.

According to Cupchik and colleagues [16], neural bases of the aesthetic experience should be studied according to the interaction between top-down attention orientation and bottom-up perceptual facilitation. In this study, researchers observed that different neural networks are the underlying foundation of a pragmatic and aesthetic orientation towards artworks and that different brain areas are activated in response to different and specific perceptual characteristics. Therefore, the aesthetic experience appears to be the result of the interaction between the personal predisposition towards artworks (i.e., top-down processes) and the perceptual inputs (i.e., bottom-up processes). Artistic ambiguity offers a good tool to test the interaction between these elements.

Perceptual ambiguity, namely the quality of perceptual stimuli of being interpreted in different ways, is one of the tricks that artists use to evoke an aesthetic experience in observers [17]. Usually, ambiguity leads to a perceptual oscillation from one concept to another, resulting in a distributed micro-consciousness, both in time and space [17,18]. Several works have studied the perception of ambiguous stimuli in artworks: among them, the research of Jakesch, Leder, and Forster [19] discovered that ambiguity can be considered a fundamental element in the aesthetic appreciation of artworks. These authors, using Magritte’s ambiguous works, showed a preference for such works over unambiguous ones, although they were more difficult to process. They also found that when looking at abstract paintings, a moderate level of ambiguity is associated with a higher level of pleasure and interest experienced [20]. According to the authors, this effect is mostly due to the involvement of a higher aesthetic pleasure. Similarly, Muth, Hesslinger, and Carbon [21] reported a strong correlation between the degree of ambiguity in artworks and aesthetic preference, where greater perceived ambiguity brought greater enjoyment and interest. This positive aesthetic evaluation has also found to be enhanced if viewers that felt able to solve perceptual problems and gain insights into the meanings of works that do not seem obvious at first Zeki [22], suggested the presence of a “cognitive ambiguity” as developed in Vermeer’s work, instilling a mystery and ambiguity in many of his paintings. The term ambiguity is here used in its neurological sense, as the certainty of many different situations or conditions, each of which has the same validity as the others. According to Zeki, the implicit behind some works of art is the result of neurological reworking and each person’s experience, adapting to different brains, at different times.

Another set of evidence comes from the study of the individual perceptual style: the perceptual style refers to individual elaboration modalities of the perceptive characteristics and it is differentiated based on “global” or “local” elaboration [23,24,25]. When elaborating the features of an artwork, individuals who have a global perceptual style—namely, those who were faster in detecting the global level of hierarchical stimuli such as Navon letters [23]—are more influenced by the context rather than the single elements, while those who have a local perceptual style—namely, those who were faster in detecting the local level of hierarchical stimuli such as Navon letters [23]—perceive the single elements rather than the context [24]. Context-related characteristics also seem to influence the aesthetic experiences of individuals in complex artworks: those who have a local perceptual style may show a different aesthetic pleasure than those who rely primarily on a global perception. It should be noted, then, that the effect of perceptual style can change throughout life: a study has shown that global perception is often observed in young rather than older individuals [26]. It could therefore be assumed that, although perceptual style influences aesthetic pleasure, its effects may change over a lifetime.

The perceptual ambiguity of Arcimboldo’s paintings differs from that of Magritte’s and Vermeer’s artworks, which can be described as a sort of “cognitive ambiguity”. It has been used in a series of studies demonstrating that perceptual ambiguity yielded to different aesthetic evaluation due to the individual’s predisposition towards the part or the whole. More in detail, individuals with local perceptual style highly appreciated Arcimboldo’s portraits and judged them as more ambiguous [27]; additionally, local prime significantly enhanced aesthetic pleasure of ambiguous portraits, whereas global prime toned down the preference of individuals with a local perceptual style for ambiguous portraits [28]. Accordingly, the content-dependent brain area of the ventral visual stream selectively activated during face perception (i.e., fusiform face area) was less activated when participants enjoyed Arcimboldo’s ambiguous portraits [13].

However, it has not yet been established whether an explanation of Arcimboldo’s ambiguity fosters individuals’ appreciation for this category of artworks. Several studies [29,30,31,32,33] examined the possible effect of the explanation of an artwork on the pleasantness expressed towards it. Russell [29] reports the “influential analysis” of Berlyne [34,35], wherein the author linked the hedonic value to three main classes of stimuli variables: collative variables (including novelty and complexity), psychophysical variables (such as color and brightness), and ecological variables (i.e., significance and associative value). Moreover, Russell [29] observed that there was an increase in the hedonic value of an artwork when paired with information that favored its interpretation and enhanced its meaningfulness. Another important contribution is made by Millis [31], who investigated the effect of different types of descriptions on the appreciation and understanding of an artwork. The results showed that, compared with the condition in which there was no description, when the titles shown simply described the scene explicitly, the perceived understanding of the work increased, although this was not the case for the corresponding aesthetic experience. These results suggested that the repetition of salient aspects of the work contributed to a more coherent representation; moreover, it may be assumed that spectators looked at the title to determine the artist’s intention and partly based their understanding on whether this intention was identified or not.

Here, we tested whether focusing individuals on salient aspects of the Arcimboldo’s artworks, namely the parts (i.e., objects, fruits) or the whole (i.e., the face) affects individuals’ aesthetic pleasure. To this aim, participants were asked to judge Arcimboldo’s artworks and Renaissance portraits before (baseline) and after (retest) a brief audio description which might focus on the parts (i.e., local descriptions), the whole (i.e., global descriptions) or on the historical background (i.e., historic description). It has been hypothesized that, in accordance with the cognitive reworking directed by the audio descriptions during the second session, there will be a difference in appreciation of the artworks between the first and the second session. Moreover, it has been expected that, during the retest session, there will be a general greater appreciation especially for the ambiguous portraits of Arcimboldo since the descriptions for the single artworks can highlight the perceptual ambiguities of the paintings themselves. Finally, it has been assumed that, in a comparison between local, global, and historical descriptions, the aesthetic appreciation of the artworks provided by respondents will be greater in correspondence of a local, rather than a global, description, and the aesthetic pleasure of the artworks provided by respondents will be greater in correspondence of a local, rather than a global and historical, description.

## 2. Materials and Methods

### 2.1. Participants

The study was conducted at the Laboratory of Experimental and Applied Psychology in “Sapienza” University of Rome (Rome, Italy). The study was approved by the International Review Board and written informed consent was obtained from participants prior to their enrolment. Inclusion criteria were ages between 18–25 years old, no history of neurological or psychiatric disorders, no substance use disorders, and no artistic expertise. Participants accepted to take part into the experiment in exchange for extra credits. According to the mentioned criteria, the final sample consisted of 86 participants (47 females and 39 males), age ranged from 18 to 25 years (M = 19.64; DS = 1.45), and age of education ranged from 13 to 16 years (M = 13.14; SD = 0.60). Sample size was determined by means of an a priori power analysis, performed using G*Power3 [23], to test repeated measures ANOVA, with an alpha of 0.05 to achieve a power of 0.95. The effect size (η_p_^2^ = 0.121) was derived from an independent sample of 10 participants comparable with the experimental group by age and schooling, who performed the pilot study. The result yielded a total sample size of 60 participants. We enrolled more than 60 participants due to possible dropout (~30%) between baseline and retest.

### 2.2. Procedure

**Figure 1:**
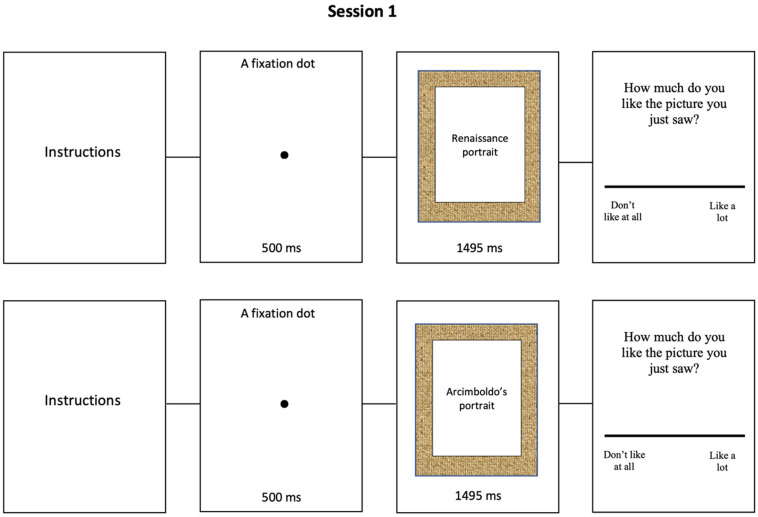
Baseline Session: Timeline and examples of stimuli.

Baseline session. Participants were asked to report the degree of aesthetic preference for each portrait through a computerized Visual Analog Scale (VAS), by placing the cursor along the VAS and confirming their answer through its left button. Trials started with the instructions, then a fixation dot of 500 ms appeared, and finally a random portrait has been presented for 1495 ms. In the final phase, on the screen appeared the written question “How much do you like the portrait you just saw?” with the VAS and to participants were asked to express their judgment for each picture, based only on their aesthetic preferences. The VAS is defined as a continuum, so the respondent is not required to provide a numerical attribution, but rather a consistent seamless gradation; furthermore, because an electronic analysis tool was used, the values are assigned automatically by a programmed algorithm [36]. VAS rating could be from “do not like at all” to “like a lot” (Figure 1).

**Figure 2:**
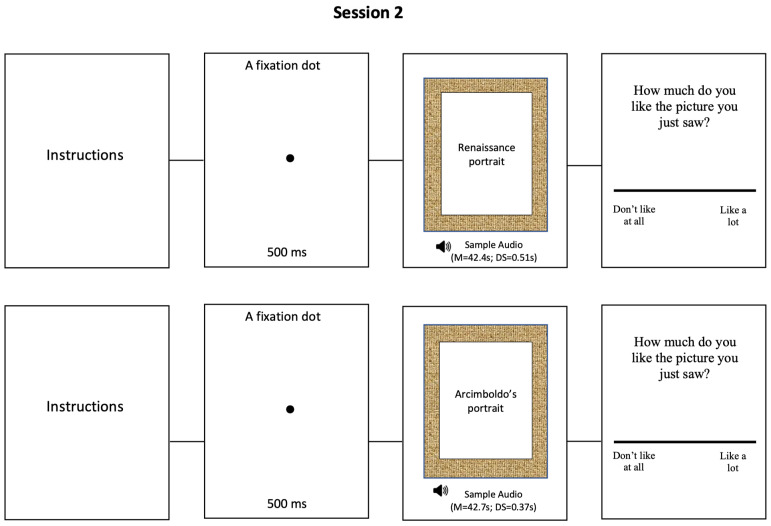
Retest Session: Timeline and examples of stimuli.

Retest session. All participants recruited in the Baseline were called up again for the next phase of the research after one week from Baseline. The stimuli set were the same of session 1 (i.e., 9 ambiguous and 9 realistic renaissance portraits). The stimuli measured 700 × 1024 pixels (width × height) and were projected in the center of the screen. Trials started with the instructions, then a 500 ms fixation dot appeared, but differently from Baseline, the random portraits were combined with a sample audio that described the portraits that the participants were watching. Participants were instructed to completely listen the sample audio and after that, were asked to evaluate their aesthetic preference degree about the portraits through VAS. When the sample audio started, the portrait remained in the center of the screen allowing participants to look carefully at the portraits and compare them with the audio description. The audio descriptions time have a mean of 42.4 s and a standard deviation of 0.51 s for the Arcimboldo’s portraits (ambiguous) and have a mean of 42.7 s and a standard deviation of 0.37 s for the Renaissance portraits (non-ambiguous; Figure 2).

#### 2.2.1. Stimuli

In both tasks, stimulus presentation and response collection were controlled by scripts running on OpenSesame [37] on a PC desktop computer. The stimuli were presented on a 27 in. computer screen (1920 × 1080-pixel resolution). The stimuli set contained 18 paintings, which can be ascribed to two categories: 9 Ambiguous paintings of Arcimboldo have been selected, consisting in portraits assembled from objects, such as animals or plants; 9 realistic Renaissance portraits, matched with Arcimboldo’s ones for gender and position of the depicted subjects. Arcimboldo’s ambiguous portraits were derived from a larger set used in previous studies [13,27,38], as those receiving a similar aesthetic evaluation of non-ambiguous Renaissance portraits. The stimuli measured 700 × 1024 pixels (width × height) and were projected in the center of the screen.

#### 2.2.2. Treatment

During the Retest session, the treatment consisted of including an audio sample describing each portrait. In particular, the portraits were described in three different forms of treatment: local, global, and historical and randomly assigned for each portrait. The description was centered on the treatment category; for example, the historical description provided information to the participants exclusively on the historical background of portrait (e.g., short description of the male or female subject, historical particulars), while the global and local descriptions provided information about the portrait in its totality (e.g., age of the subject, information about their posture) or in its details (e.g., embroidery of clothes for unambiguous artworks, reference to the objects that form the portrait for ambiguous ones), respectively. The descriptions of ambiguous and Renaissance portraits were developed in a double-blind procedure by two independent psychologists with an expertise in art history (for more detailed information about the description, see Appendix A).

### 2.3. Data Analysis

Statistical analyses were performed using Statistical Package for Social Science (SPSS; version 25.0; IBM SPSS, Armonk, NY, USA). Descriptive statistics were performed for gender, age, and years of education. 

Responses occurring before 500 ms and after 5000 ms were discharged from the analysis to exclude too fast and no “gut-feeling” responses. Then, for each participant and category (i.e., Arcimboldo and Renaissance portrait) the mean VAS score and mean response time (RT) were computed and used in subsequent analyses. Two repeated measures ANOVA, with a 2 × 2 × 3 factorial design (session × category × treatment), was performed on VAS and RT scores. Statistical significance in post hoc pairwise comparisons was determined using Bonferroni correction (as implemented in SPSS).

## 3. Results

**Figure 3:**
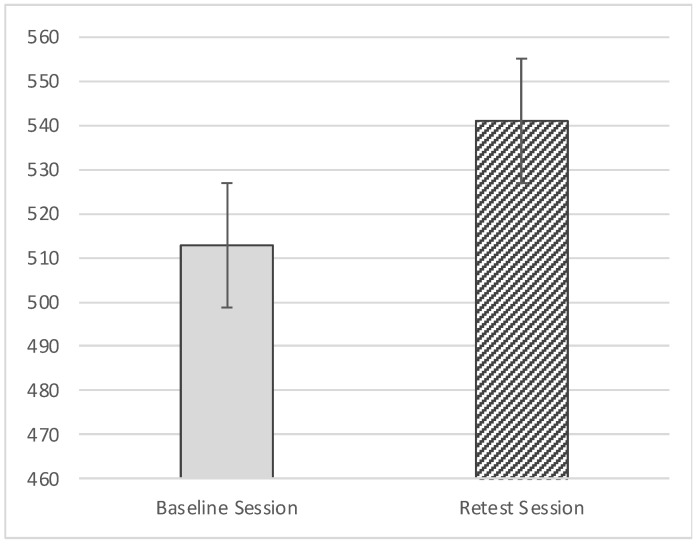
Effect of Session on Visual Analogue Scale (VAS).

**Figure 4:**
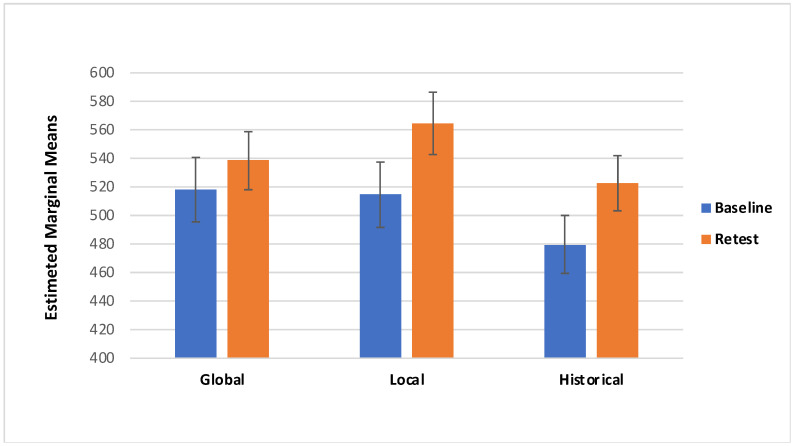
Visual Analogue Scale (VAS) for the ambiguous portraits in both Baseline and Retest session for category.

**Figure 5:**
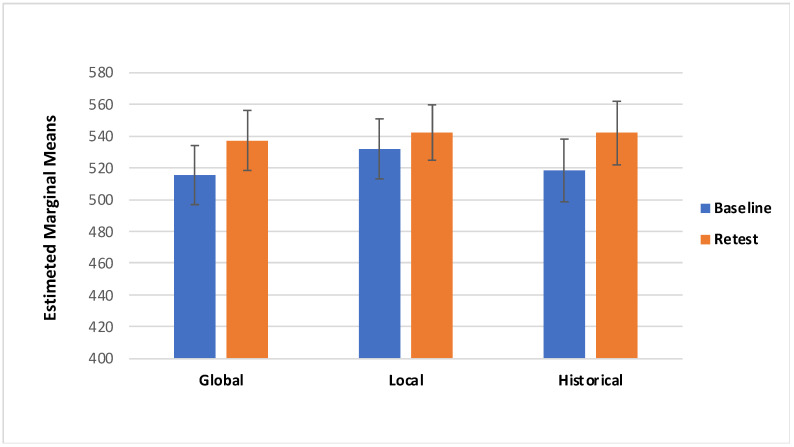
Visual Analogue Scale (VAS) for the non-ambiguous portraits in both Baseline and Retest session for category.

Concerning the VAS score there was a significant effect of Session (F_(1,85)_ = 11.775, *p* = < 0.05; effect size: η_p_^2^ = 0.122): specifically, participants showed higher VAS scores in the retest session (M = 541.00; SE =11.53) compared with the Baseline (M = 512.90; SE = 11.20; Figure 3). Additionally, there was a marginally significant interaction effect of Session*Category*Treatment (F_(2157)_ = 3.050, *p* = 0.054; effect size: η_p_^2^ = 0.035; Greenhouse–Geisser-corrected due to departure from sphericity); with local and historical treatments of ambiguous artworks showing higher scores during retest session (local M = 564.30, SD = 201.43; historical M = 522.26, SD = 179.37) than the baseline, and global and historical treatment of non-ambiguous artworks showing higher scores in the retest session (global M = 537.15, SD = 172.49; historical M = 541.87, SD = 184.97) than the baseline (Table 1, Figure 4 and Figure 5).

**Figure 6:**
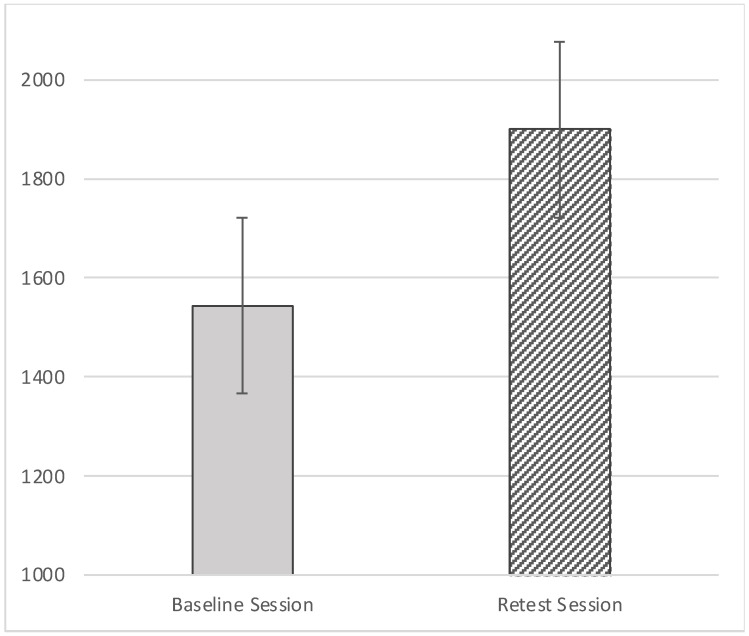
Effect of Session on Response Time (RT).

**Figure 7:**
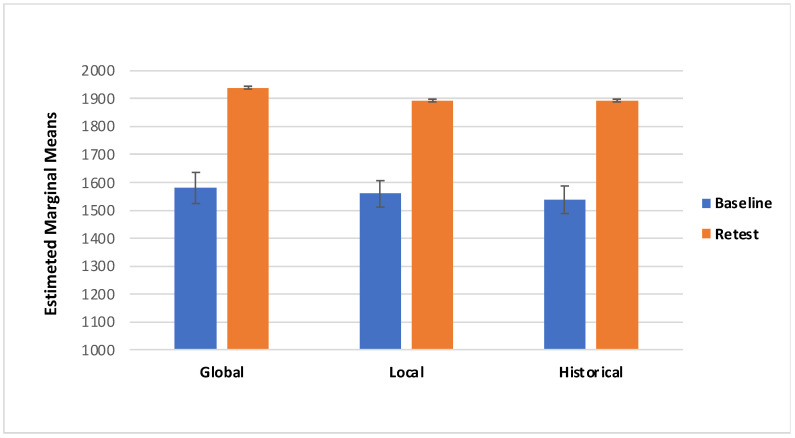
Response Time (RT) for the ambiguous portraits in both Baseline and Retest session for category.

**Figure 8:**
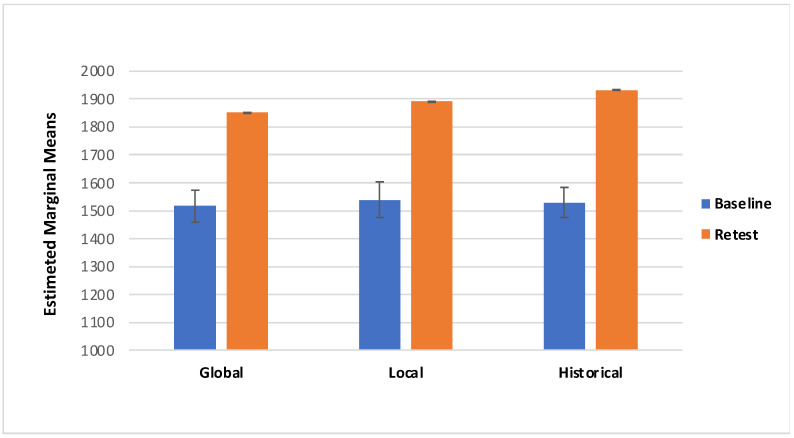
Response Time (RT) for the non-ambiguous portraits in both Baseline and retest session for category.

Concerning the RT, there was a statistically significant effect of the Session (F_(1,85)_ = 39.754, *p* = < 0.001; effect size: η_p_^2^ = 0.319): participants were slower during the retest session (M = 1899.30, SE = 65.20) than the baseline (M = 1544.01, SE = 44.96; Figure 6, Figure 7 and Figure 8).

## 4. Discussion

The main aim of this study was to evaluate the effect of the descriptions on the aesthetic pleasure of the portraits. The literature reports that description has a positive effect on the pleasure that comes from looking at an artwork [29]. The same effect has been found on the effect of title of the artworks; specifically, an abstract painting accompanied by an elaborative title could be easily understood [30,31,33]. However, the understanding did not increase their hedonic value, per se [30]. According to the theory of the “effort after meaning” [39], the pleasure derives from the perception of having correctly interpreted the artwork and the artist’s message. Belke and colleagues [40] showed that the awareness of artwork’s characteristics (e.g., technique, stylistic features, and compositional elements), could enhance their appreciation. According to these premises we have implemented three kinds of treatment to investigate the effect of specific descriptions on ambiguous and non-ambiguous portraits. The primary aim of our study was to identify any differences in aesthetic appreciation following a description of the artwork; for this reason and for ambiguous portrait characteristics, the treatments have been classified: global description, local description, and historical description (i.e., control condition). The findings from the present study suggest that aesthetic preference was influenced by the description of a piece of artwork. First, we found that descriptions provided during retest yielded to higher VAS scores (Figure 4 and Figure 5); additionally, RT was slower during the retest (Figure 7 and Figure 8). Taken together, these results suggest that the descriptions may affect the aesthetic evaluation likely due to a better knowledge of the painting; also, longer RT, we detected during retest, might mirror a more accurate artwork reading. Second, even if marginal, the interaction we detected between the type of treatment and the category of artwork deserves further consideration, considering previous studies.

Previous studies suggest that context-related characteristics could affect aesthetic experience [15]. Additionally, the study by Cupchik and colleagues [16] suggests that the aesthetic evaluation arises from the interaction between top-down and bottom-up processes affecting attention and perceptual facilitation. Consistent with these premises, individuals who have a local style highly appreciated Arcimboldo’s portraits and evaluated them as more ambiguous [13]. Similarly, local prime seems to enhance aesthetic appreciation (i.e., local prime yielded higher rate) of ambiguous portraits compared with the global prime [28]. Consistent with these previous studies, here we found that local treatment selectively fostered the aesthetic appreciation of Arcimboldo’s artwork. On the contrary, global description fostered appreciation of non-ambiguous portraits, different to Arcimboldo’s. These results, taken together, support the idea that an interplay occurs between characteristics of the paintings (e.g., ambiguity) and individual’s predisposition towards the artwork. In the more general context of the aesthetic theories, we may speculate that the effects we observed here occur later in the aesthetic processing. According to the Chatterjee’s idea [14], visual information is processed step by step during the aesthetic experience, starting from the first visual perception of the artwork, which is processed in the same way as other visual objects, up to the attentional processes, which redirect the processing towards the salient visual properties; finally, these properties are modulated by the attentional network, feedback, and feed-forward processes, also involving emotional systems. Several studies suggest that understanding an artwork results in an activation of the rewarding system in the brain [41,42] and solving the perceptual problems conducts in self-rewarding feelings. Moreover, it could be hypothesized that the effect we found here occurs at the later stages of the processing steps proposed by Chatterjee [14], consistent with results on response times, which are slower during retest. Indeed, the more expertise a perceiver acquires, the more differentiated and more rewarding aesthetic experiences might be. The importance of top-down knowledge was discussed by several studies suggesting that the amount of information (e.g., explicit information) about the portrait affects aesthetics experiences, conducting in an “elaboration effect” that helps to find a meaning and reduces uncertainty of the portrait [43,44,45]. The aesthetic experience is a process that requires time; in fact, visual processing and cognitive mastery of the artwork lead to an aesthetic emotion and evaluation, resulting in a positive affective state change and leading to pleasure, satisfaction, and a motivation potential [46].

## 5. Conclusions

In sum, we found that aesthetic pleasure was higher during the retest than the baseline, suggesting that the description we provided enhanced the aesthetic pleasure of artworks. Interestingly, the local and the global treatments seem to have opposite effects on Arcimboldo’s and Renaissance portraits. Arcimboldo’s were judged better following the local descriptions, whereas Renaissance portraits were judged better following global descriptions. The increase we detected in the response times suggests a more accurate and detailed reading of the artwork, because of the treatment we provided between baseline and retest. Taken together, these findings suggest that aesthetic preference was affected by the description of a piece of artwork, likely due to a better knowledge of the painting which prompts a more accurate (and slower) reading of the artwork, with opposite effects of local and global descriptions on ambiguous and non-ambiguous portraits, consistent with the idea that aesthetic pleasure arises from the interaction between top-down orientation of attention and bottom-up perceptual facilitation [16].

The perceptual ambiguity of Arcimboldo’s artwork provides a good tool to test our experimental hypothesis without spurious perceptual effects. However, it limits any possible generalization of our results to other types of perceptual complexity and ambiguity in art. Furthermore, the uniqueness of the stimuli used in our study prevents any possible generalization of our results to other categories of artworks. In accordance with these considerations, it would be interesting to conduct similar studies with different sets of ambiguous paintings.

Further studies should test the effect of mere exposition to Arcimboldo’s artwork, without auditive stimuli as a control condition; it would be interesting to assess whether the participant’s perceptual style plays a role in the degree of aesthetic pleasure and reaction time in the evaluation; moreover, the possible influence of third variables (e.g., familiarity, repeated measures, etc.) could be assessed through a new study comparing two different homogeneous samples. In addition, functional imaging studies could be conducted to identify the regions and neural mechanisms underlying the observed effects.

## Figures and Tables

**Table 1:** Pairwise comparisons.

Pairwise Comparisons Session * Category * Treatment						
Category	Treatment	Session		Mean Difference	Std. Error	Sig. ^b^
Ambiguous	Global	2	1	20.355	13.277	0.129
	Local	2	1	49.759	15.273	0.002
	Historical	2	1	42.964	14.184	0.003
Renaissance	Global	2	1	21.771	8.912	0.017
	Local	2	1	10.300	11.245	0.362
	Historical	2	1	23.440	9.470	0.015

Note. Based on estimated marginal means; b. Adjustment for multiple comparisons: Bonferroni. * Interaction between different conditions.

## Appendix A

### Appendix A.1. Description of “Ambiguous” Portrait “Spring” Created by Arcimboldo, 1573

#### Appendix A.1.1. Historical Treatment

According to some art experts, the paintings of “Four seasons” and the “Four elements” were a celebration of Emperor’s dominion over both the seasons (macrocosm) and the elements subordinated to them (microcosm). All the members of each pair were meant to face one another according to the relationship that associates seasons, elements but also age; in this example “Spring” was characterized by allegorical elements of youth. The changing of seasons refers to the succession of the emperor, underlying his ability to dominate and lead the whole creation.

#### Appendix A.1.2. Global Treatment

We are looking at a bust-length portrait of a young woman facing to the left side. This is a portrait of “Spring”, represented as a woman in her youth, featured as a vigorous and lively character. The subject has pinkish cheeks, and the shape of the lips shows their smoothness while revealing a shiny smile. The young woman is wearing a green dress with a white collar and a brooch on her chest. The elegance of both the dress and the headgear probably represents her noble origin.

#### Appendix A.1.3. Local Treatment

We are looking at a still-life painting consisting entirely of spring plants and flowers. Looking from the bottom-up visual, a green area is presented, consisting of the foliage of several plants. In the middle of the portrait there is a bundle of small white flowers, such as daisies and jasmine, which settle on different petals of all shades of pink which overlap each other, creating a spring carpet. On this, flowers with a larger corolla and brighter colors stand out. At the top, there is a multicolored bouquet from which a white lily emerges.

### Appendix A.2. Description of “Non-Ambiguous” Portrait “Federico da Montefeltro” Created by Piero della Francesca, 1467–1472

#### Appendix A.2.1. Historical Treatment

Federico da Montefeltro was a Duke, Earl, and Lord of many Renaissance palaces. A political activist and a historical enemy of Lorenzo the Magnificent, he has transformed the Duchy of Urbino into the one of the most important artistic and cultural sites in Italy, attracting painters, architects, and artists; for this reason, he is remembered as one of the main benefactors of the Renaissance. He is always depicted in profile (left side) to hide the loss of the eye caused by a spear shot; in fact, he was called “the Scarred Duke”.

#### Appendix A.2.2. Global Treatment

We are faced with a portrait of a man on the left profile. The background is filled with a naturalistic landscape that fades as far as the eye can see. In this context, the subject in the foreground, who is dressed in a deep red dress and headgear, seems to merge with it. The light and tones of the landscape are clear and warm and seem to blend with the protagonist of this portrait.

#### Appendix A.2.3. Local Treatment

The dark complexion of the subject stands out, compared with the landscape, with his face framed by black and shaggy hair. The painting has many details both in the man and in the landscape. The face is marked by deep wrinkles on the cheeks and around the eyes and skin imperfections; the nose’s profile is unnaturally crushed. In the background appears a water basin with moving boats, ploughed fields, roads, villages with towers and castles and a mountain range on the horizon.
